# Supplementary figures and images for: Anti-obesity compounds, Semaglutide and LiPR, and PrRP do not change the proportion of human and mouse POMC+ neurons
Source: PLoS One. 2025 Aug 13;20(8):e0329268. doi: 10.1371/journal.pone.0329268 (PMC12349008; doi:10.1371/journal.pone.0329268)

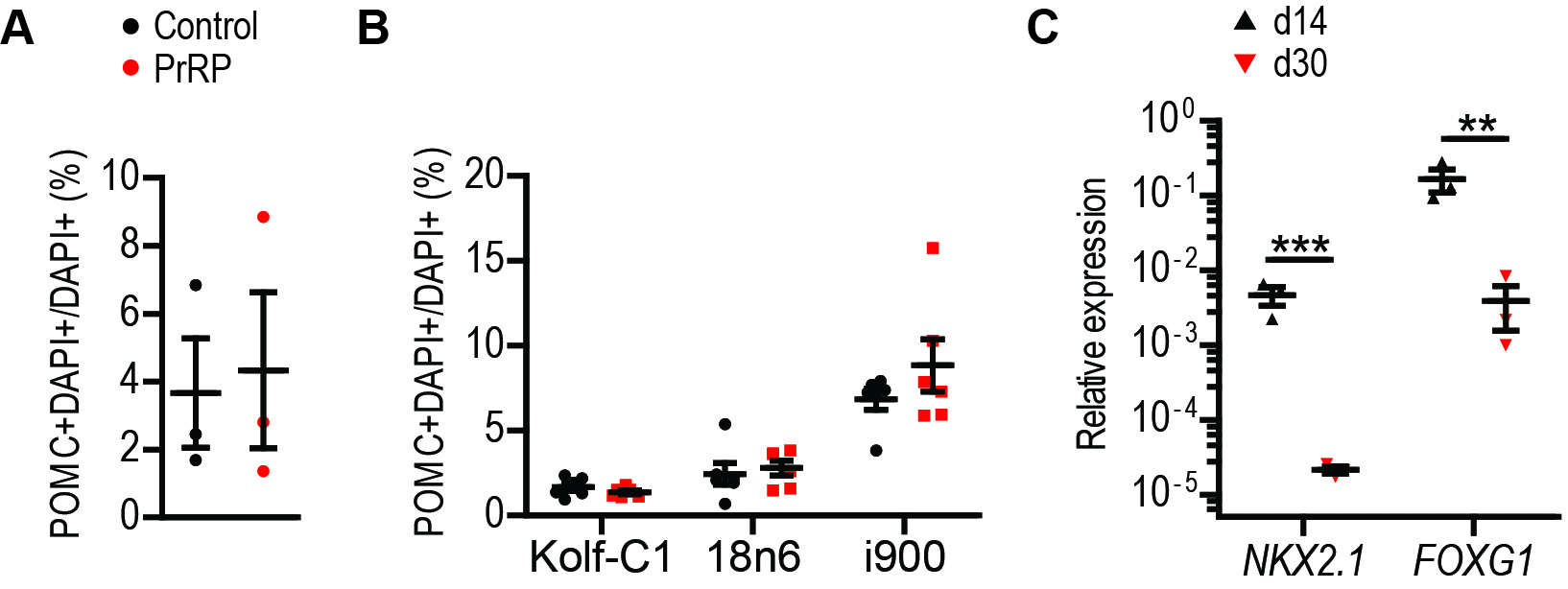

Supplement: S1 Fig — (A) Quantification of the proportion of POMC+DAPI + /DAPI+ cells of Control and PrRP-treated hiPSC-derived neurons at day 30. (B) Proportion of POMC+DAPI+ cells per hiPSC line (individual data points represent technical replicates). (C) RT-qPCR expression of NKX2.1 and FOXG1 at day 14 and 30 from Control and PrRP-treated hiPSC-derived neurons. n = 3 hiPSC lines. Two-tailed T-Test: **p < 0.01, ***p < 0.001. Data are presented as mean ± SEM. (TIF) [file pone.0329268.s001.tif]
