# Supplementary material for: Anti-obesity compounds, Semaglutide and LiPR, and PrRP do not change the proportion of human and mouse POMC+ neurons
Source: PLoS One. 2025 Aug 13;20(8):e0329268. doi: 10.1371/journal.pone.0329268 (PMC12349008; doi:10.1371/journal.pone.0329268)
Supplement: Attachment — Submitted filename: Response to Reviewers PONE-D-24-58169.pdf [file pone.0329268.s003.pdf]

Response to Reviewers

PONE-D-24-58169

Title: "Anti-obesity compounds, Semaglutide and LiPR, do not change the proportion of human and mouse POMC+ neurons"

Journal Requirements:

When submitting your revision, we need you to address these additional requirements.

1. Please ensure that your manuscript meets PLOS ONE's **style requirements**, including those for **file naming**.

Answer: We revised the manuscript and adjusted the subheadings, figure references, and text to style requirements and file naming of PLOS ONE.

2. To comply with PLOS ONE submissions requirements, in your Methods section, please provide additional information regarding the experiments involving animals and ensure you have included details on (1) **methods of sacrifice**, and (2) **efforts to alleviate suffering**.

Answer: In Methods section, we included details on (1) methods or sacrifice, and (2) efforts to alleviate suffering. The new details are included on lines 261-264 (new text highlighted in red font): "After 21 days of treatment, animals were sacrificed by pentobarbital overdose followed by a complete exsanguination, transcardially perfused with 4% PFA and brain isolated as described before (26). The total anaesthesia was used to nullify suffering."

3. Please provide a complete Data Availability Statement in the submission form, ensuring you include all necessary access information or a reason for why you are unable to make your data freely accessible. If your research concerns only data provided within your submission, please write "**All data are in the manuscript and/or supporting information files**" as your Data Availability Statement.

Answer: We provided a complete Data Availability Statement in the submission form. We deposited all raw data related to all figures in our manuscript. The data were deposited to a public data depository, OSF, approved by PLOS ONE. The data has following DOI: https://doi.org/10.17605/OSF.IO/84ZXK This DOI is now linked to the manuscript.

4. We note that the grant information you provided in the '**Funding Information**' and 'Financial Disclosure' sections do not match. When you resubmit, please ensure that you provide the correct grant numbers for the awards you received for your study in the 'Funding Information' section.

Answer: We provided correct grant numbers to match information provided in the 'funding information' and 'financial disclosure'.

5. We note that you have included the phrase "**data not shown**" in your manuscript. Unfortunately, this does not meet our data sharing requirements. PLOS does not permit references to inaccessible data. We require that authors provide all relevant data within the paper, Supporting Information files, or in an acceptable, public repository. Please add a citation to support this phrase or **upload the data** that corresponds with these findings to a stable repository (such as Figshare or Dryad) and provide and URLs, DOIs, or accession numbers that may be used to access these data. Or, if the data are not a core part of the research being presented in your study, we ask that you **remove the phrase** that refers to these data.

Answer: The data related to the phrase "data not shown" are not a core part of the research being presented and the phrase was removed.

6. Please review your **reference list** to ensure that it is complete and correct.

Answer: We reviewed our reference list to ensure that it is complete and correct.

Review Comments to the Author:

Reviewer #1: This study investigated the effects of PrRP, LiPR, and semaglutide on the POMC neuron maturation, but did not find any significant effects of these drugs. The authors need to revise the title because LiPR appears only in Figure 4. They may consider deleting specific drug names or include PrRP as well.

Answer: We included PrRP in the manuscript title as suggested by the reviewer. The new title is "Anti-obesity compounds, Semaglutide and LiPR, and PrRP do not change the proportion of human and mouse POMC+ neurons".
